# Musical coordination in a large group without plans nor leaders

**DOI:** 10.1038/s41598-020-77263-z

**Published:** 2020-11-23

**Authors:** Louise Goupil, Pierre Saint-Germier, Gaëlle Rouvier, Diemo Schwarz, Clément Canonne

**Affiliations:** 1 STMS UMR 9912 (CNRS/IRCAM/SU), Paris, France; 2 University of East London, London, UK

**Keywords:** Psychology, Human behaviour

## Abstract

A widespread belief is that large groups engaged in joint actions that require a high level of flexibility are unable to coordinate without the introduction of additional resources such as shared plans or hierarchical organizations. Here, we put this belief to a test, by empirically investigating coordination within a large group of 16 musicians performing collective free improvisation—a genre in which improvisers aim at creating music that is as complex and unprecedented as possible without relying on shared plans or on an external conductor. We show that musicians freely improvising within a large ensemble can achieve significant levels of coordination, both at the level of their *musical actions* (i.e., their individual decisions to play or to stop playing) and at the level of their *directional intentions* (i.e., their intentions to change or to support the music produced by the group). Taken together, these results invite us to reconsider the range and scope of actions achievable by large groups, and to explore alternative organizational models that emphasize decentralized and unscripted forms of collective behavior.

## Introduction

It is traditionally believed that, as group size increases, so does the tendency for inertia^1^, social loafing^2^, irrational decisions^3^, or “groupthink”^4^. Larger groups also seem to be more prone to coordination loss^5^: while group performance may sometimes increase with the number of agents involved in disjunctive problem solving, or in collective decision-making involving verbal deliberations^6^, such group performance is more likely to decrease in tasks requiring tight coordination between group members, because of the higher number of “interaction links” at play^7^. As a result, large groups engaged in joint actions that require a high level of flexibility and adaptability are generally taken to be unable to successfully coordinate on their own without additional resources such as sufficiently specific shared plans that are common knowledge among the participants^8^, or leaders that supervise and guide the interactional dynamics at play within the group^9^.

This is no less true in the case of music performance^10^. It is well known that music can be played in large ensembles, involving a dozen up to a 100 participants, such as symphonic orchestras, jazz big bands, and batucada or gamelan ensembles. But in such cases, the increased difficulty of coordination is typically met by specific properties of the situation, such as the existence of explicit and fine-grained shared plans of the music to be played (e.g., a score, a set of oral instructions, etc.), and/or a functional and hierarchical organization of the ensemble (e.g., a conductor and section leaders, instrumental sections with different roles, etc.). In that perspective, it has been shown that conductors’ movement kinematics greatly influence communication and coordination among orchestra members, and, more generally, that the functional and hierarchical organization of an orchestra plays a crucial role in how the information flows from conductor to musicians, and from one instrumental section to the other^11–13^. As for musical scores and collaborative rehearsing, they typically allow orchestra members to establish (at least partially) shared plans and shared performance goals, which then enable them to develop internal models of their partner’s parts and to reliably predict each other’s actions^14^.

In a relatively small number of cases, large ensembles have taken up the challenge of coordinating without conductors, such as the *Persimfans* in the early Soviet Union or the *Orpheus Chamber Orchestra*; but in those cases, orchestra members can still count on the existence of a shared plan, in this case a score specifying the contribution of each member. Conversely, in some improvised musical practices such as *Soundpainting*^15^ or *Conduction*^16^, large groups of musicians typically perform without a preexisting score; but their coordination then heavily relies on the presence of the conductor who is assigning in real time different roles or actions to the musicians based on a pre-established code.

Overall then, large groups of musicians appear to solve coordination problems either by relying on detailed and explicitly agreed upon shared plans (e.g., a score), or by establishing a hierarchy between the group members, or at least a minimal functional organization within the group. One can then wonder if large musical ensembles could function at all without these resources, and create music that is, at least to some extent, comparable in its complexity to the music produced by large ensembles in more usual settings, without falling back on simplistic and/or homogenous collective behaviors, such as systematic imitation or isochronous playing^17^.

Collective free improvisation (henceforth CFI) seems to provide a perfectly adjusted framework to address this question. First, in CFI, performers deliberately avoid relying on shared referents^18^, such as chord changes or melodic scales. While free improvisers arguably have the overarching shared goal of creating complex and unprecedented music together, this highly underspecified shared goal hardly amounts to a shared plan, in the sense that it does not entail any specific sequence of individual or collective actions, not even a loosely defined one. In other words, the issue of how to *temporally* and *qualitatively* organize the individual and collective musical behaviors on shorter and longer time scales in a given performance remains in its entirety. Second, free improvisers generally strive to maintain an egalitarian organization which is not constrained by traditional instrumental functions^19^, while aiming at displaying behaviors that are both highly differentiated and yet intertwined^20^. Thus, CFIers deliberately refuse to establish a pre-determined hierarchical structure that could facilitate coordination within the ensemble.

Yet, even expert improvisers have raised serious doubts concerning large-groups’ ability to coordinate efficiently during CFI in order to produce satisfactory musical outcomes. For instance, Simon Fell highlights the “overlooked difficulties” of playing in “large-scale ensembles: actually hearing what other players are doing can be difficult if they are physically separated from you by a large number of musicians. This can limit the potential for subtle interaction to only those musicians who are relatively near, or encourage musicians to play more loudly or forcibly than they would otherwise consider”^21^. Jacques Siron is even harsher in his description: “everyone plays a lot, but without really committing themselves, interrupting and resuming without reason; no clear decision appears; listening and attention are distracted, uneven; the overall sound is opaque, confused, shapeless; it quickly becomes impossible to clearly perceive what each person is playing, except for the noisiest instruments; the general form is devoid of any angles or articulations”^22^. And double-bassist Joëlle Léandre concurs, stating that she does not “like big ensembles […]. I don’t believe in masses, I believe in the intimacy of listening.[…] In improvisation, which is a natural and urgent music, duos are the most perfect ensemble”^23^. Thus, many improvisers tend to construe CFI as being mainly a small-group affair, and to question the ability of large groups to coordinate efficiently.

In line with this view, previous empirical research on collective improvisation have been exclusively focused on small groups, typically duos or trios^24–29^, leaving us largely in the dark as to whether large groups of musicians can achieve coordination at all while freely improvising.

There is however a recent and growing tendency within the world of improvised music of performing freely improvised music in very large ensembles—as witnessed by the examples of the *Orchestre des Nouvelles Créations, Expérimentations et Improvisations Musicales* (Paris), the *Splitter Orchestra* (Berlin), the *Insub Meta Orchestra* (Geneva), the *Glasgow Improvisers Orchestra* (Glasgow), or the *London Improvisers Orchestra* (London)—in which typically more than fifteen musicians freely improvise together, without the help of an external conductor or a pre-established plan. We took advantage of the existence of such orchestras to conduct an experiment on coordination in large-group free improvisation, and asked the *Orchestre des Nouvelles Créations, Expérimentations et Improvisations Musicales* (ONCEIM hereafter for short) to participate in a study. Having played together for the past 10 years, the ONCEIM is now well-known for their extended collective free improvisations involving 15 to 30 musicians. Crucially, while the ONCEIM has developed over the years a sense of its own sonic identity that partially reduces the sheer unpredictability of their performances^30^, the common ground thus provided to the musicians is more akin to a global aesthetic framework than it is to a full-fledged plan that would constrain the temporal unfolding of their performances. As one member of the orchestra puts it: “When I’m introducing a new sonic event in the ONCEIM, I never know what will happen. Even though we know each other very well, the reaction will always be different because of the number of musicians involved” (quoted in^30^). The ONCEIM also displays a very fluid organization: the exact line-up of an ONCEIM performance can widely vary from one performance to the other, depending on the availability of each musicians, and the ONCEIM routinely performs with only 15 to 20 musicians of the 34 official members of the group; placements within the orchestra change from one session to the other in order to avoid the sedimentation of pseudo-instrumental sections; and although pianist and improviser Frédéric Blondy acts as an “external ear” for the orchestra, providing feedback to the musicians after rehearsals and concerts^30^, he does not play within the ONCEIM nor does he “conduct” in any way the orchestra, which plays in a fully “unsupervised” fashion. For all these reasons, the extended improvisations performed by the ONCEIM offer a perfect case study for investigating how orchestra-sized groups can deal with *unplanned* and *unconducted* collective improvisation.

Now, coordination is notoriously tricky to investigate in CFI^31^. CFI crucially differs from more familiar genres of improvised music such as bebop or even free jazz in the sense that it lacks a definite tonal grammar and is generally not pulsed. In such a complex musical context, with an astounding variety of instrumental and timbral expressions, it does not make much sense to assess coordination by relying on the simple metrics of temporal synchronization, timbral proximity or harmonic coordination that are traditionally used in studies examining musical coordination (e.g., in^32^). We thus need to assess coordination on a more abstract level. In the following, we assume that there is musical coordination when musicians interact—i.e., when their intentions, decisions and actions “mutually […] affect one another’s”^33^—, and when such interactions are geared towards a specific outcome (some overall effect or some specific group behavior) that is successfully achieved. More precisely, we operationalize this abstract notion of coordination in large-group CFI by focusing on two fundamental aspects of collective music-making: *musical actions*, considered at their most basic level (i.e., whether to play or to remain silent) and *directional intentions* (i.e., whether to change the music of the group or to support/maintain it).

First, we ask how musicians collectively organize their musical actions. As Wilson and MacDonald^26^ put it, “when improvising music as freely as possible within a large ensemble, deciding whether to play or remain silent at points in a performance remains a fundamental musical choice”. We thus examine whether improvisers’ musical actions are inter-dependent (i.e., suggesting that musicians mutually affect one another), and whether the interactions happening on this level are geared towards a certain group behavior, such as turn-takings or keeping the number of “active” musicians under a certain threshold at any given moment.

Second, we examine the extent to which musicians agree on their *intentions* regarding the general directionality to give to the ongoing performance. While musicians’ intentions and representations may diverge considerably in CFI^26,34,35^, sustained contradictory intentions regarding a feature as fundamental as the temporal evolution of the music are likely to prevent improvisers to make music together at all^36^. We thus ask whether musicians’ decisions on this level of changing vs. supporting are inter-dependent, and whether musicians tend to agree on such *directional intentions*, therefore allowing for the group to “navigate” into the performance’s sonic stream in a cohesive way.

The current study aimed at examining whether musicians coordinate at these two levels. But we were also interested in investigating the individual and relational factors that might explain how improvisers spontaneously organize their interactions with one another in such large groups. In particular, we hypothesized that, in an orchestral setting, musicians could cluster their interactions according to a variety of factors. First, coordination might be modulated by the spatial localization of musicians within the group: are musicians positioned near from one another more coordinated? Are musicians positioned in the periphery of the orchestra less coordinated with the others? Second, musicians might cluster according to the kind of instrument they play: are musicians playing identical or similar instruments more coordinated? Third, differences in coordination among musicians might be explained by socio-musical criteria: Are musicians used to playing with one another in this kind of setting more coordinated? Are musicians with a higher expertise in CFI more coordinated with the others?

The secondary aim of our study is thus to assess the respective relevance of these different factors in an explanation of the interactional dynamics at play in a free improvisation orchestra.

## Methods

### Participants

16 musicians from the ONCEIM participated in our experiment (2 women, mean age = 39.9 years ± 7.5 SD). All were highly-skilled musicians actively involved in the CFI local scene. Ten musicians were members of the ONCEIM since its foundation in 2011 (8 years of participation), and 6 musicians joined the ONCEIM at a later date (average years of participation = 4.7 ± 1.8 SD, min = 2 max = 7). The composition of the orchestra was the following: 6 strings (3 double bass; 2 altos; 1 violin); 6 winds (1 clarinet; 3 saxophones; 1 trumpet; 1 euphonium); 2 electronics; 2 drums. One musician participated in the experiment but had to be excluded from the analysis because she did not play at all. In addition, a technical error prevented us from recording slider data for three additional musicians. All participants signed an informed consent. All participants signed an informed consent to participate in the study, as well as a consent for publication of identifying information or images in an online open-access publication.

### Procedure and design

Musicians were asked to freely improvise for circa 20 min, without any additional constrains. Their entire performance (of duration 19′27′′) was video-recorded (the recording, slightly edited to introduce a fade in and a fade out, is available at https://youtu.be/7DzW1061P54). While the musicians were playing, an expert listener (the ONCEIM “artistic director”) was asked by the researchers to segment the performance in real-time, by writing down the timings of the main formal articulations between the different parts of the improvised performance. This resulted in a segmentation of the improvised piece into 8 sequences of mean duration 144.87 s ± 60.21 SD (from the first to the last sequence: 225; 45; 135; 130; 215; 135; 95; 179 s).

**Figure 1:**
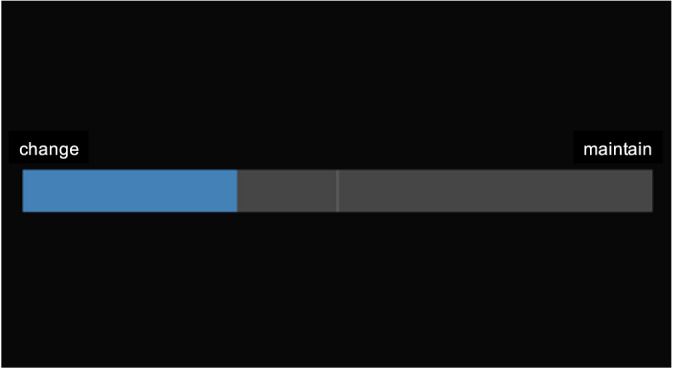
Screenshot of the tactile digital slider used by the musicians to annotate their performance while listening back to it. 16 musicians were asked to rate the extent to which they were trying to change, or on the contrary to support, the music produced by the group.

Immediately after the performance, and before having time to debrief the performance, the ONCEIM members were asked to provide a continuous annotation of their performance as follows: they were seated in front of a large screen on which we projected the audio–video recording of the performance. Each musician was provided with a touch-screen iPod and asked to indicate, as the recording was playing, what her *directional intention* was at each time of the improvisation by using a digital slider. The interface was developed with the *soundworks* architecture^37^. Musicians could move between one extremum representing the intention to *change the direction of the music collectively produced by the group*, and another representing the intention to *support the direction of the music collectively produced by the group* (see Fig. 1). Musicians were told that they could use every intermediate slider position, with the middle position corresponding to a “neutral” intention, with respect to the change vs support opposition. Importantly, they could not see each other’s ratings. Note that our measure of *directional intentions* thus relies on a post-hoc annotation, with the obvious limitation that it may be contaminated by thoughts that occurred after the performance, and involves memory reconstruction. Yet, this is the only way to investigate improvisers’ intentions in this complex setting in a non-invasive fashion, given that online measures (e.g., relying on MIDI pedals during the performance, see^38^) result in strong perturbations of the ongoing performance, which is far from ideal to assess whether musicians can indeed coordinate in such a complex setting.

For each of the musicians, we also assessed how familiar she was with each of the other musicians by asking her to list all of the musicians with whom she had never played before (familiarity rating of 0), rarely played with (familiarity rating of 1) or frequently played with (familiarity rating of 2) outside of the ONCEIM. These ratings were averaged for each pair of musicians to assess their level of familiarity. We then also averaged these familiarity scores at the individual level in order to obtain for each musician an index of her familiarity with others, measuring her overall degree of musical familiarity with the members of the ONCEIM. Mean familiarity with others was 0.65 ± 0.34 SD. We also measured musicians’ degree of expertise in improvising in large ensembles as the average between the number of years they had been engaging with the ONCEIM (M = 6.75 years ± 1.95 SD) and the average of years they had been practicing CFI (M = 17.1 years ± 6.8). Mean expertise was 11.9 years ± 3.88 SD. We also assessed each musician’s degree of spatial eccentricity within the orchestra by measuring how many musicians separated her from each of the other musicians, and averaging this value at the individual level. Lower values thus reflect higher centrality, and higher values higher eccentricity. Mean spatial eccentricity was 1.76 ± 0.74 SD. Spatial proximity was also estimated for each pair as the number of musicians separating them from each other. Finally, we grouped musicians into three instrumental families (winds, strings, and inharmonic including drums and electronics). For each pair, we then computed a binary index depending on whether they belonged to the same (1) or different (0) instrumental families. We thus had four individual factors for each musician (expertise, spatial eccentricity, instrumental family and familiarity with others) as well as three relational factors for each pair (spatial proximity, instrumental proximity and familiarity).

### Data analysis

**Figure 2:**
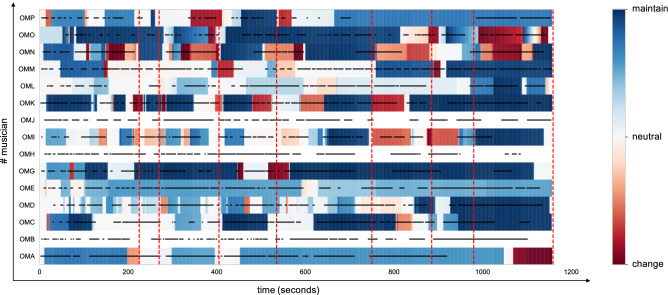
Raw data. The y-axis corresponds to individual musicians, and the x-axis represents time in seconds. Colors show musicians' reports about their *directional intentions* upon re-listening to the performance following the collective improvisation. Black horizontal lines show the *musical actions* of each musicians. Red vertical dotted lines show the boundaries of the musical sequences identified by the expert annotator. Data for the directional intentions of OMB, OMJ and OMH are missing due to a technical error.

Data were analyzed in *python* with the *scipy* and *statsmodel* packages. We identified from the video and audio recordings each of the musicians’ *musical actions*, defined as phases were the musician was actually playing as opposed to phases where the musician remained silent. *Musical actions* were coded by four (C.C., L.G., G.R., P.S.G.) of the authors, who had considerable experience with the music of the ONCEIM, allowing to discriminate individual musicians’ contributions from the sound of the ensemble. This coding was achieved with a resolution of 1 Hz, which is precise enough to characterize the music played by the ONCEIM, that often consists in slowly developing textures and changes (see video example). Slider data (i.e., directional intentions) were registered with a resolution of the millisecond, but resampled to 1 Hz to allow comparison with *musical actions*. All digital sliders were automatically synchronized with the video file projected to the musicians so that we could directly compare slider ratings with *musical actions*. Slider ratings (a value between 0: change the music and 1: support the music) and musical actions (0/1) were then analyzed in very similar ways. Figure 2 shows the evolution of each improviser’s *directional intentions* and *musical actions* over time.

As explained above, coordination between the musicians was investigated by looking both at the improvisers’ *musical actions* (whether they are playing or remaining silent) and at their *directional intentions* (the degree to which they are trying to change, or on the contrary to support, the music).

For each possible pairs of musicians within the orchestra (N = 15 musicians so 105 pairs for *musical actions*; N = 12 musicians so 66 pairs for *directional intentions*), we estimated how the two musicians mutually influenced each other by estimating whether each improvisers’ *musical actions* and *directional intentions* forward predicted each of the other improvisers’ intentions using *Granger Causality*. Over the past few years, Granger causality has become a standardized way to assess information flow between pairs of agents engaged in collective music-making (e.g., see^11,13,27,39^). Here, we computed Granger causality between the real values of each pair of musicians, but also between their time-scrambled values, allowing us to estimate divergence from chance with a bootstrapping procedure. For each pair of musicians, Granger causality between the two vectors was computed using the stats model toolbox in Python^40^. To fit the assumptions of Granger causality, vectors were checked for stationarity, and de-trended if necessary. The maximum lag of the model—which was selected over the whole dataset by choosing the value that minimized both AIC and BIC, following an established procedure^11,41^—was 16 time points. The two values of causality (musician A causing musician B and musician B causing musician A) were averaged for each pair to estimate *causal density* within the pair. This metrics reflects the extent to which musicians’ intentions and actions relate to each other, while allowing for the fact that one musician may slightly precede the other. Thus, this metric reflects the degree of interdependency, or interaction, between the two musicians.

Next, we computed two further measures for each dimension to assess whether these interactions reflect coordination, i.e., whether they appear to be geared towards some specific group behavior. We first describe how we computed these metrics to assess musicians’ coordination at the level of *musical actions*, before describing how we assessed coordination at the level of *directional intentions*.

First, we computed for each possible pairs of musicians how *correlated* their *musical actions* were, by computing Spearman correlations between the real values for these pairs, but also between their time-scrambled values. This *correlation* reflects the extent to which musicians’ musical actions match, and are synchronous (e.g., there is a positive correlation if when musician A decides to play, musician B also generally decides to play). Now, although we expect a significant association between musicians at this level, it needs not be positive: it is likely that within the orchestra, some musicians tend to play at the same time (leading to a significant positive correlation), while other musicians tend to engage in turn taking (leading to a significant negative correlation). However, an absence of coordination between the musicians would be observed if there is no significant correlation whatsoever between the timings of their *musical actions*. Thus, we also measured *sonic organization* as the absolute value of Spearman’s rho. This metric reflects coordination in that it measures the extent to which any two improviser’s musical actions are organized into a consistent and systematic pattern, regardless of the direction of their association. Again, to assess whether it differed from chance level, this metric was computed for real and time-scrambled values.

Second, for *directional intentions*, we computed the average *distance* between each pair of musicians’ slider values. This *mean slider distance* was first computed at each time point and then averaged across time, resulting in one value per pair. Again, this metric was computed for real values, and time-scrambled values, to allow us to estimate chance-level. For each pair, the *mean slider distance* reflects how close the two musicians’ directional intentions are. We also computed a complementary measure of *correlation* (Pearson’s rho), reflecting the extent to which musicians’ directional intentional match, and are synchronous (e.g., there is a positive correlation if when musician A decides to change the music, musician B also generally decides to change the music). This measure was computed for both real and time-scrambled pairs. The two metrics above (mean slider distance and Pearson’s rho) give us an indicator of musical coordination, because they reflect the extent to which a pair of musicians agrees about the direction that the group as a whole should take.

Finally, in order to investigate more global group behaviors and have a sense of their evolution over time, we also computed three metrics at the level of the group as a whole. First, we computed the percentage of musicians who decided to play for every second by averaging our *musical actions* variable (0: not playing; 1: playing) over all the musicians. Second, we computed the mean slider values at each second, to have a sense of the direction that was the most popular in the group at each second. Third, we computed the overall degree of alignment of *directional intentions* within the group at each time point as follows: *group alignment* = *absolute value of (the number of musicians intending to support the music, with a slider value* > 0.5*—number of musicians intending to support the music, with a slider value* < 0.5*)/number of musicians*. Group alignment thus reflects the overall convergence between musicians regarding their *directional intentions* (do they tend to “push” the music in the same direction or not?), with values of 1 attained when all musicians display identical intentions, and values of 0 attained when the group splits into two equal halves displaying opposite intentions. This measure was computed for real combinations of musicians, as well as for fake combinations of musicians (time-scrambled values) to estimate the chance-level. We also averaged these values separately in time windows centered around the middle, or the junction of the musical sequences identified by the expert annotator. Time windows encompassed the duration ranging from minus to plus 16 s around the formal points (middle or junction), which was the time window that was found to minimize the AIC in the Granger causality analysis described above, which resulted in 256 time points for the middle of the sequences (8 sequences by 16 time points on either side) and 256 time points for the junctions.

The impact of familiarity, expertise, centrality and instrumentation on coordination was assessed in hierarchical mixed regressions. We report beta estimates, standard errors, t-values (with Satterthwaite approximations to degrees of freedom), p-values and chi-squares for hierarchical nested model comparisons with likelihood ratio tests, computed with the lme4 and lmerTest packages in R^42^.

### Ethical approval and consent

Ethical approval for this study was obtained at INSEAD/Sorbonne University Center for Behavioural Science, Paris, France. All methods were carried out in accordance with their guidelines and regulations. All participants signed an informed consent.

## Results

### Musicians’ musical actions and directional intentions are interdependent

**Figure 3:**
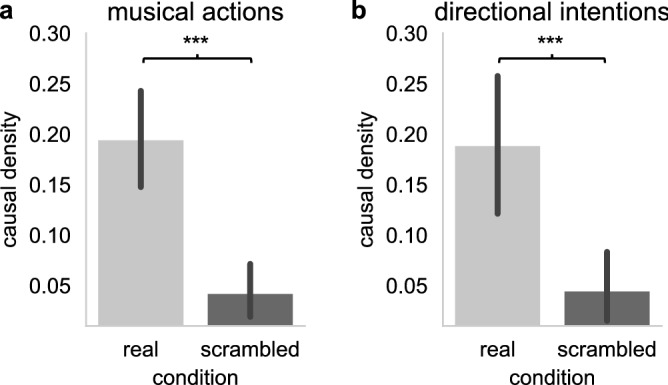
Causal density between pairs of musicians for musical actions and directional intentions. Causal density (Granger causality averaged across the two musicians) between real and time scrambled pairs of musicians’ *musical actions* ((**A**), left) and *directional intentions* ((**B**), right). Error bars show 95% confidence intervals, and black asterisks show the pairwise t-test comparing real vs. scrambled data with *** corresponding to p < 0.001.

Despite the complexity and the density of the sonic landscape they created in their performance, which could arguably impact the performers’ ability to swiftly react to one another or even to precisely hear each other, improvisers’ *musical actions* (i.e., their decisions to play or not), as well as their *directional intentions* to support or change the music, were highly interdependent. Causal density between pairs of improvisers’ *musical actions* highly significantly differed from chance-level (Fig. 3A, real pairs’ causal densities for *musical actions*: M = 0.195 ± 0.26 SD; time-scrambled pairs: M = 0.043 ± 0.14 SD, t(104) = 5.15, p < 0.001, d = 0.72), and the same result was obtained for *directional intentions* (Fig. 3B, real pairs M = 0.189 ± 0.27 SD, time-scrambled pairs: M = 0.045 ± 0.145 SD; t(65) = 3.6, p < 0.001, d = 0.67). Thus, musicians’ intentions to change or support the music, as well as their decisions to either play or remain silent had an impact on the evolution of the other musicians’ own intentions and actions. This result shows that musicians interacted during the performance, with mutual influences occurring within pairs of musicians as the performance unfolds. Now, in itself, this is not enough to be able to claim that musicians’ actions and intentions were coordinated. Thus, we asked next whether these interactions are organized in specific ways that suggest that they are geared towards a certain group effect or joint outcome.

### Improvisers’ musical actions are coordinated

**Figure 4:**
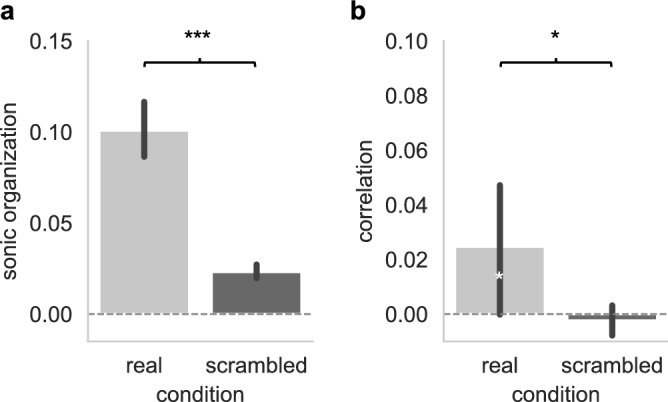
Coordination of musical actions. (**A**) The degree of sonic organization within the orchestra was measured as the absolute value of the correlation (Spearman’s rho) between pairs of musicians’ musical actions (playing versus not playing). Sonic organization highly significantly differed from chance level (t(104) = 9.69, p < 0.001, d = 1.35). (**B**) Correlations (Spearman’s rho) significantly differed from the chance-level computed from scrambled pairs (t(104) = 2.06, p = 0.042, d = 0.29) and from zero (t(104) = 1.99, p = 0.049, d = 0.19), although with a small effect size reflecting the fact that some pairs tended to be correlated, and other pairs to be anti-correlated. Error bars show 95% confidence intervals, black asterisks show the pairwise t-test comparing real vs. scrambled data (with ***p < 0.001) and white asterisks the one-sample t-tests comparing Spearman’s rho to zero (with *p < 0.05).

To assess whether improvisers *musical actions* were coordinated, we examined whether their degree of *sonic organization* (i.e., the mean absolute value of Spearman’s rho) significantly differed from chance (estimated from time-scrambled data). We found that pairs of improvisers’ *musical actions* were more closely associated than what would be predicted by chance (see Fig. 4A, real pairs’ mean absolute value of Spearman’s rho: M = 0.1 ± 0.08 SD; time-scrambled pairs: M = 0.02 ± 0.02 SD, t(104) = 9.69, p < 0.001, d = 1.35), showing that improvisers arranged their musical actions in relations with one another in a consistent fashion. Examining the directionality of the *correlation* between pairs of musicians revealed that this was due to correlations as well as anti-correlations. While improvisers’ musical actions were positively correlated on average (see Fig. 4B, real pairs’ correlation: M = 0.025 ± 0.13 SD), this was barely different from chance-level (time-scrambled pairs: M = − 0.002 ± 0.03 SD, t(104) = 2.06, p = 0.042, d = 0.29). The reason for this is that while 37 pairs (35.24%) showed a significant positive correlation, 27 pairs (25.71%) showed anti-correlated *musical* *actions*. Thus, while some pairs tended to play at the same time, other pairs tended to avoid playing at the same time, thus engaging instead in turn-taking behavior. Note also that a substantial number of pairs (41 pairs, 39.05%) showed no significant associations, which is consistent with the idea that in a big ensemble like the ONCEIM, not every pair of musicians interacts during the performance. Below we investigate several factors that may determine whether improvisers’ *musical actions* associate or not during the performance.

**Figure 5:**
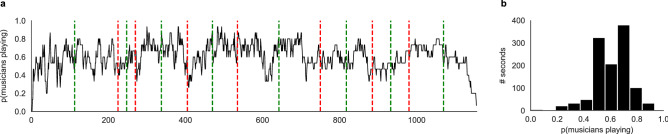
Musical actions at the group level. (**A**) The percentage of musicians playing at each second was estimated by averaging our *musical action* variable at each time point. (**B**) Histogram showing the distributions of the percentage of musicians playing at each second. Red dotted lines show the junctions between musical sequences, determined by the expert annotator. Green dotted lines show the middle of each musical sequence.

The overall pattern in which these dyadic interactions results appears more clearly when looking at how musical actions are organized at the level of the group. Figure 5A shows the evolution of the overall *musical actions* of the group, that is, the percentage of musicians who actually decided to play at each time point. What is remarkable, here, is that musicians were never playing all at the same time, and, as can been seen in Fig. 5B, that they never reached the maximum value (mean % of musicians playing overall = 60.6% ± 15 SD, range = [0 93.33]), and only reached the minimum value at the end of the performance. This suggests that they were aiming to keep the number of active musicians around a certain threshold, probably to avoid creating an overly saturated musical space, while allowing for everyone in the group to actually produce sounds at some point during the performance. Thus, even without pre-planification or predetermined hierarchical organization, musicians were able to spontaneously coordinate their sonic behaviors to organize into musical turn-takings, exchanging their musical positions (i.e., playing vs. remaining silent) with each other in a coherent way to limit the number of musicians playing at the same time.

### Improvisers’ directional intentions are coordinated, but only at specific times during the performance

**Figure 6:**
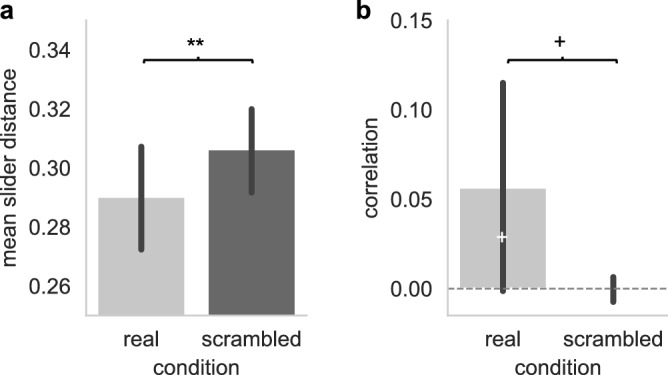
Coordination of directional intentions. (**A**) The degree of congruence between pairs of musicians’ directional intentions was measured as the average distance between slider values. Mean slider distance for real pairs highly significantly differed from mean slider distances computed over time-scrambled pairs (t(65) = 2.98, p = 0.004, d = 0.25), indicating that musicians’ directional intentions were closer to each other than would be predicted by chance. (**B**) Correlations (Pearson’s rho) only marginally differed from the chance-level computed from scrambled pairs (t(65) = 1.86, p = 0.067, d = 0.33) and from zero (t(65) = 1.85, p = 0.068, d = 0.23), with a small effect size reflecting the fact that some pairs tended to be correlated, and other pairs to be anti-correlated. Error bars show 95% confidence intervals, black asterisks show the pairwise t-test comparing real vs. scrambled data (with **p < 0.01; ^+^p < 0.07) and white asterisks the one-sample t-tests comparing Pearson’s rho to zero (with ^+^p < 0.07).

To assess whether musicians’ ratings changed in congruent directions (i.e., aligned), we examined whether the correlation between their ratings over time, and the mean distance between their ratings overall, significantly differed from chance (estimated from time-scrambled data). Regarding the mean distance between the ratings given by pairs of musicians, we found that pairs of musicians’ intentions were closer from each other on the sliders than what would be predicted by chance (see Fig. 6A, real pairs’ distance in ratings averaged over time: M = 0.29 ± 0.07 SD; time-scrambled pairs: M = 0.31 ± 0.06 SD, t(65) = 2.98, p = 0.004, d = 0.25). The results regarding correlation were far less clear cut. Musicians intentions were positively correlated on average (see Fig. 6B, real pairs’ correlation: M = 0.06 ± 0.25 SD), but this was only marginally different to chance (time-scrambled pairs: M =  − 0.0004 ± 0.03 SD, t(65) = 1.86, p = 0.07, d = 0.33). A closer examination of the pairs suggested that correlation was not systematic in between pairs: 31 pairs (47%) showed a significant positive correlation, and 22 pairs (33%) showed anti-correlated intentions, while 13 pairs (20%) showed no significant association.

**Figure 7:**
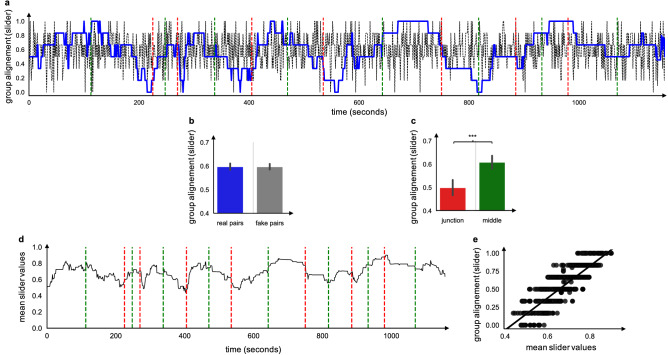
Directional intentions at the group level. (**A**) Group alignment was estimated for the directional intentions reported on the sliders at each second. Blue lines show the real values of group alignment, and grey dotted line group alignment computed over time scrambled data. Red dotted lines show the junctions between musical sequences, determined by the expert annotator. Green dotted lines show the middle of each musical sequence. (**B**) Comparison with chance. Group alignment for real pairs (blue) and time scrambled pairs (grey). (**C**) Evolution. Group alignment restricted to time windows centered around junctions (red) or the middle of the musical sequences (green) identified by the expert annotator. (**D**) “Group intention”, estimated at each second by averaging the values reported on the sliders across musicians. (**E**) Correlations between group average slider values vs. group alignment for slider values. We show the best fitting regression line, which was linear: this reflects the fact that agreement was typically related to an increased intention to support rather than to change the music. Each dot shows an individual time point.

Thus, overall there was limited evidence that pairs of musicians’ intentions regarding the evolution of the music were consistently coordinated over the whole performance. Now, if a substantial number of pairs of musicians “pushed” in contradictory directions (i.e., some want to change the music while the others want to support it), this can hardly count as a case of coordination. However, this correlation measure reflects the convergence of intentions over the whole duration of piece. Given the sheer complexity of the interactions taking place in CFI, and the high unpredictability of the sonic environment created by the improvisers, one should probably not expect improvisers’ intentions to be indifferently coordinated at all times of the performance. Coordination in CFI might be of a more local nature, with the musicians’ intentions significantly aligning only at some specific phases of the performance. To investigate this hypothesis, we turned to our global measure reflecting the alignment of intentions at the level of the full group and analyzed its temporal evolution. Figure 7A shows that an interesting pseudo-oscillatory pattern emerged in the evolution of the alignment of musicians’ intentions, which led alignment to not differ from chance overall (Fig. 7B, mean alignment in the group computed over the whole performance = 0.598 ± 0.25 SD vs. 0.599 ± 0.22 SD for time scrambled data, t(1157) =  − 0.08, p > 0.9). Thus, rather than aligning continuously, musicians’ intentions were only aligned at certain points during the performance. Crucially, alignment corresponded to the middle of the musical sequences identified by the expert annotator (the musical director of the ONCEIM): as can be seen on Fig. 7C, musicians’ intentions were significantly more aligned during the central parts of the sequences (mean alignment during time windows centered around the middle of each musical sequence = 0.61 ± 0.24 SD) as compared to during the articulation phases between two adjacent sequences (i.e., mean alignment during time windows centered around the middle of each musical sequence = 0.5 ± 0.27 SD, t(254) = 4.86, p < 0.001). Furthermore, we observed that in such moments of high alignment, musicians were largely on the “support” side of their sliders. As shown in Fig. 7E, there was a strong linear relationship between average values on the slider and group alignment (Spearman’s rho = 0.85, p < 0.001). Thus, when they tended to align, musicians were generally attempting to support the music.

This suggests that coordinating intentions is especially important when musicians perceive a need to consolidate the music after a phase of transition. This is consistent with the analysis of improvisers’ descriptions offered by Wilson and MacDonald^35^, who observed that improvisers’ accounts were often convergent in passages in which all of them were engaged in some form of “maintaining”, so to speak. And this further suggests that, in creative and challenging contexts such as large-group improvisation, local coordination might be enough to keep the group going. Overall, the results show that, even without pre-planification or a predetermined hierarchical organization, improvisers interacting within such a large group can locally coordinate their intentions to stabilize given musical situations within the complex and rapidly changing sonic environment of the performance.

### Familiarity between pairs of musicians predicts stronger coordination of intentions, but spatial or instrumental proximities do not

After having established that ONCEIM members could achieve some degree of coordination on both the level of their *musical actions* and on the level of their *directional intentions*, we tried to identify which relational factors predicted “stronger” coordination between any two players. To do so, we ran hierarchical linear mixed regression analysis with our four metrics of coordination as dependent variables (*correlation of intentions*, *correlation of actions*, *mean slider distance* and *sonic organization*), and our three relational factors of familiarity, instrumental proximity and spatial proximity as independent variables. The outputs of these models are reported in Table 1.

**Table 1:** Associations between coordination at the level of intentions and relational factors.

	Familiarity	Instrumental proximity	Spatial proximity
Correlation			
Beta	0.058	0.05	0.02
se	0.04	0.067	0.019
t-value	1.34	0.73	1.09
p-value	0.1	0.5	0.28
Mean slider distance			
Beta	− 0.023	− 0.012	− 0.0056
se	0.013	0.02	0.005
t-value	− 1.83	− 0.06	− 1
p-value	0.037*	0.57	0.32

We report the output of linear mixed regressions assessing the impact of pairs of musician’s familiarities, and spatial and instrumental proximities on the correlation and mean distance of their ratings on the sliders. Significant effects are highlighted in bold. None of these regressions were significant for musical actions (all p-values > 0.05).

Regarding *directional intentions*, we found that musicians’ familiarity with each other was significantly related to the coordination of their intentions: musicians that were more familiar with one another provided ratings that were closer to each other on the sliders (i.e., reduced mean distance, beta =  − 0.023 ± 0.012 sem, t =  − 1.83, p = 0.037). However, we did not find any significant associations between spatial or instrumental proximity and any of the coordination metrics. This result suggests that rather than associating based on spatial proximity within the orchestra, or on the basis of instrumentation, ONCEIM musicians cluster on a more abstract level, so to speak, which reflects both social and aesthetic proximity. While familiarity was the only ‘relational’ factor related to how well pairs of musicians coordinate within the orchestra, other factors at the individual level predicted how much musicians coordinated with their peers, as we describe below.

The similar regression with *musical actions* revealed no significant associations between our measures of coordination and individual factors (all p-values > 0.05). By contrast, we found significant associations with individual factors, as we describe below.

### Relationship between individual factors and coordination with others

Turning to individual factors, we analyzed whether expertise, general familiarity with other musicians, the instrument played by the musician and her spatial position within the orchestra predicted how strongly each musician coordinated with the other musicians. To do so, we ran hierarchical linear mixed regression analysis for each dimension (musical actions and directional intentions) separately, with each of our two metrics of coordination for these two dimensions as dependent variables, and our four individual factors of familiarity with others, expertise, instrumental family and spatial eccentricity as independent variables.

As shown in Table 2 were the full output of the models is presented, we observed some variability depending on musical instruments: string players were less coordinated with others as shown both by lower *sonic organization* and higher *mean slider distance* in this instrumental group. Regarding expertise, at the level of *musical actions*, expertise was negatively associated with sonic *correlation*, suggesting that expert musicians tended to engage in turn-taking with other musicians more (and thus to play a key role in the regulation of the overall sonic activity of the group). Spatial eccentricity negatively impacted how coordinated improvisers’ *musical actions* were: increased eccentricity was associated with lower *sonic organization*. Finally, and consistent with our observation at the level of pairs, we found that higher familiarity with other musicians was significantly associated with a smaller *mean distance* to others’ slider ratings, as well as a higher *correlation* with the *musical actions* of others. This means that improvisers that are musically well-acquainted with many of the ONCEIM members—i.e., musicians who occupy a central position within the network of socio-musical relationships that comprise the ONCEIM—play a cardinal role in ensuring the ensemble’s cohesion, by providing more robust coordination links within the ensemble. Musicians that are more familiar with others interact with their fellow improvisers in a more consistent and coordinated fashion, thus creating a pole of (relative) stability in the ever-changing interactional dynamics at play in this large-group setting.

**Table 2:** Associations between coordination at the level of intentions and actions and individual factors.

	Directional intentions				Musical actions			
	Expertise	Familiarity	Eccentricity	Instrument	Expertise	Familiarity	Eccentricity	Instrument
Correlation								
Beta	0.003	0.06	0.06	w/i: − 0.11 w/s: − 0.15 s/i:0.04	− 0.006	0.065	− 0.02	w/i: 0.005 w/s: 0.01 s/i: − 0.008
se	0.01	0.096	0.054	w/i: 0.09 w/s: 0.1 s/i:0.11	0.002	0.02	0.01	w/i: 0.019 w/s: 0.022 s/i: 0.022
t-value	0.3	0.6	1	w/i: − 1.3 w/s: − 1.96 s/i: 0.34	− 2.4	2.8	− 1.5	w/i: 0.27 w/s: 0.55 s/i: − 0.33
p-value	0.78	0.09	0.4	w/i: 0.24 w/s: 0.19 s/i: 0.74	0.04*	0.02*	0.15	w/i: 0.8 w/s:0.6 s/i:0.74
Sonic organization								
Beta					− 0.001	0.017	− 0.023	w/i: 0.017 w/s: − 0.04 s/i: 0.06
se					0.002	0.023	0.01	w/i: 0.016 w/s: 0.02 s/i: 0.02
t-value					− 0.39	0.757	− 2.12	w/i: 1 w/s: − 1.97 s/i: 2.8
p-value					0.2	0.54	0.028*	w/i: 0.3 w/s: 0.08 s/i: 0.02*
Mean slider distance								
Beta	0.0011	− 0.06	− 0.05	w/i: − 0.005 w/s: 0.094 s/i:0.099				
se	0.003	0.03	0.013	w/i: 0.02 w/s: 0.024 s/i:0.027				
t-value	0.345	− 1.825	− 3.895	w/i: − 0.25 w/s: 3.84 s/i: − 3.6				
p-value	0.73	0.02*	0.12	w/i: 0.8 w/s: 0.008** s/i: 0.01**				

We report the output of linear mixed regressions assessing the impact of individual musicians’ familiarity with others, spatial eccentricity, instrumental family (winds, strings of inharmonic) and expertise on the correlation and mean distance of their ratings on the sliders (intentions) with those of other musicians, and correlation and degree of sonic organization of their musical actions with other musicians. Significant effects are highlighted in bold. For instruments, the first row shows the comparison between winds and inharmonic instruments, the second row shows strings vs. winds and the bottom row shows strings vs. inharmonic instruments.

## Discussion

Our study puts the belief that large groups are unable to engage in sophisticated and unsupervised improvised actions to a test, by empirically investigating coordination within a large group of musicians performing collective free improvisation—a genre which is especially demanding in terms of coordination, musicians having to spontaneously react and adapt to a highly unpredictable, and often rapidly changing, sonic environment. Here, we show that, despite the absence of an explicit shared plan, or of an external conductor, musicians freely improvising within a 16-piece ensemble can achieve significant coordination both at the level of their *musical actions* (i.e., their individual decisions to play or to stop playing tend to be interdependent, to influence each other, and to collectively keep the number of active musicians around a certain threshold) and at the level of their *directional intentions* (i.e., their intentions to change or to support the music produced by the group tend to be interdependent, to influence each other, and to align, although only locally).

Such results should invite us to reconsider the range and scope of actions achievable by large groups. While small-scale dyadic interactions and “modest sociality”^8^ account for a significant part of our everyday lives, one of the most striking aspect of our social behavior is the capacity we have to do things in large groups involving dozens of people, from traditional collective rituals^43^ to team-based collaborative research^44^. Group size certainly impacts the way agents tend to behave and the temporal unfolding of their joint action^9^. In particular, swift and spontaneous coordination might be more difficult to achieve in large groups than in comparatively smaller groups, as emergent coordination mechanisms such as perception–action couplings and affordances^45^ probably tend to become less and less efficient within larger groups, where each agent has only access to a limited part of the overall perceptual scene, and in which a greater heterogeneity of individual motor repertoires is to be expected. Similarly, it might be more difficult for large groups’ members to resist entrainment effects, such as the increasing speed of clapping among audience^46^, and thus to display flexible or autonomous behaviors.

But this does not mean that individual agents cannot be trained to successfully improvise together within a large group. In that perspective, it is worth noting that Frédéric Blondy, ONCEIM’s “artistic director”, describes the very first improvisations of the ensemble as a “huge mess, escalating louder and louder very quickly” (personal communication to C.C., January 7th 2016). In other words, musicians had to learn over the years how to adapt their individual behaviors to fit this new “orchestral” situation, by developing specific improvisation strategies, such as limiting the number of musicians simultaneously interacting at any given point of the performance or, as can be heard on the recorded performance, reducing the degree of unpredictability by playing continuous sounds or repeated figures. In a way, then, musicians adapted their playing to reduce the difficulty of the highly challenging coordination problem raised by large-group CFI. However, it is important here to distinguish such strategies—that provide regulative collective guidelines or secondary performance goals—from pre-determined plans which would specify each of the agents’ actions beforehand and determine the precise unfolding of each of the agents’ actions as a score would. Similarly, although roles might emerge over time (e.g., some musicians may keep playing short and contrasting sounds while other may keep producing continuous textures), these roles remain implicit and highly sub-determined, as compared to the fixed functional organization found in traditional symphonic orchestras or in gamelan ensembles^47^.

According to Canonne^30^, ONCEIM musicians also had to reinforce important improvisation skills, and in particular listening skills, learning to swiftly navigate within very dense and complex sonic environments and to commit their auditory attention to selected aspects of the overall texture. This tendency to aurally “scan” the orchestra in order to search for privileged points of interaction appeared in our study, in that we observed that ONCEIM members do not interact with each other indifferently. Perhaps unsurprisingly, familiarity seems to play a key role in explaining interactions within the orchestra. ONCEIM members tended to preferentially interact with the musicians that they independently had some kind of familiarity with—musicians they feel related to on a social or aesthetical level. This is consistent with previous findings showing that free improvisers who were used to playing with each other tend to develop similar mental models (i.e., they tend make similar mappings between sound-types and action-types), thus making coordination between them easier^48^. More generally, ONCEIM members interacted more closely with improvisers that occupy a central position within the complex network of musical relationships that comprise the ONCEIM, i.e., musicians that were used to perform with a large number of ONCEIM musicians outside of the specific context of the ONCEIM. They thus relied on social proximity to select their interactions rather than on spatial proximity, which is often taken to be cardinal in large-group coordination^9^. This may be due to the distal nature of sounds, and to the difficulty there is to precisely localize in space numerous entangled sounds in a complex musical context. Further empirical studies could investigate whether this priority of social proximity over spatial proximity in explaining the distribution of interactions within a large group of agents engaged in collective improvisation is due to the specificity of the musical medium or if it extends to other kinds of complex improvised joint actions.

This aspect is reminiscent of a broad musicological literature which emphasizes the importance of social relations in music performance, and particularly in improvised music^49^. First, interpersonal relationships between the improvisers are not only the support of the musical performance; there are also its primary material, what is explored and played with by the musicians through their sonic exchanges (see^27^ for similar insights). As Clarke^50^ (p. 174) puts it, free improvisation has “at least as much to do with an exploration of interpersonal dynamics as it [has] to do with a direct manipulation of musical materials”. Second, in the absence of a musical script prescribing how improvisers are supposed to behave, the pre-existing socio-musical relationships that exist among musicians act as a kind of surrogate script, a social script, with familiarity and network centrality strongly affecting the sonic interactions taking place between the improvisers during the performance. To quote Clarke^50^ (p. 175) once again, “in free improvisation, […] a certain social context is established […] and the musical interactions are then a consequence of the nature of these social relationships”.

Even if we showed here that musicians *can* coordinate without plans nor leaders in large groups, showing *how* exactly they are able to do so would require to design more controlled experiments. A possibility might be that shared local goals—such as “changing the musical direction”, “intensifying the music”, or “ending the performance”—actually emerge in the course of the performance through the perception of salient events^51^, and that such local goals support coordination^52^. However, it remains an open question whether the coordination mechanisms involved in large groups of improvisers are the same than in comparatively smaller groups; in particular, it becomes less and less likely that some given sonic event could afford group members similar local goals as the number of agents involved increase^26^. In our study, we found that coordination was following an oscillating pattern, from phases of (quasi-) unanimous intentional alignment to phases of strong intentional divergence. This result substantiates previous descriptions of musical behavior in CFI offered by Borgo^53^ or Canonne and Garnier^54^, who describe CFI performances as being similar to complex dynamical systems^55,56^, alternating between phases with fixed attractors and phases without such attractors. If this analogy is correct, local coordination in large groups of improvisers may simply emerge from basic interactional rules (e.g., imitation, contrast, or indifference), modulated by individual parameters such as the cognitive load and the boredom of the performer^54^, rather than being the result of more sophisticated intentional mechanisms; this issue could be addressed in future work by investigating the relative contribution of each of these factors, as well as determining whether poles of strong agreement or disagreement constitute specific attractor states.

This oscillatory pattern also means that, in this kind of context at least, coordination between agents is not continuous, but rather local. That being said, even if continuous coordination were possible (which is highly unlikely given the complexity of the coordination problem improvisers have to face in such a large group), it might not be desirable. As Sawyer^57^ puts it, improvised performances are “problem-finding processes” rather than “problem-solving” ones. In that perspective, group dissensus is not only a difficulty that the group has to overcome; it is also something to work with, a resistance upon which the musicians can exert their creativity to dynamically bring the performance in unexpected musical directions. More generally, freely improvised performances are instances of creative joint actions, in which agents aim at creating complex and unprecedented musical products: in such creative actions, too much synchrony and behavioral alignment might be negatively associated with aesthetic appeal^58^, which may also explain why ONCEIM musicians tended to avoid continuous coordination.

While improvisers do not need to be coordinated with each other all the time, they need to coordinate at least sometimes. The regularity of such moments of strong group coordination might be even more crucial in the context of large groups, which may be more prone to dislocation. It is striking that, in our study, changes (i.e., moments of transitions between two adjacent sequences, as perceived by our expert annotator) were not so much the result of unanimously shared intentions as they were the result of a higher divergence within the group. On the contrary, improvisers’ intentions were maximally aligned when attempting to *support* or *maintain* a given direction shortly after a phase of discoordination. This pattern of behavior suggests the existence of something like a repair mechanism, musicians’ coordination significantly strengthening at strategic points during the performance, precisely when the group is on the verge of imploding under the weight of its internal contradictions and needs to find a new stable collective behavior.

Some years ago, Kamoche et al.^59^ challenged us to look *“beyond the jazz metaphor”* and explore alternative models for organizational improvisation—a trend in management and organizational theory which (mainly) draws on improvised artistic practices to suggest frameworks that are meant to fit innovation-oriented teams or teams evolving in unpredictable environments, thus requiring a high degree of flexibility and velocity. Jazz is certainly a popular paradigm when it comes to urge team managers to trust the creativity of their teammates and allow them to collaborate in a more autonomous fashion^60,61^, and some multinationals are even eager to compare themselves to “a jazz band, not a symphonic orchestra”^62^. But the popularity of the jazz metaphor might also be explained by the fact that jazz is more continuous than it would first appear with traditional models of team organization; after all, jazz groups still obey a functional organization and follow shared sets of instructions as a basis for their performances. In this regard, CFI, with its highly decentralized and unscripted approach, provides a more radical departure from the planification-centered and hierarchically-structured models of team organization. While CFI is sometimes presented as a quasi-utopian model for “the creation of new, unexpected, and productive cocreative relations among people”^63^, such broad claims seem closer to a catchy slogan than to an empirically falsifiable hypothesis. By evidencing the possibility of effective coordination in a large-scale group during free improvisation, our study provides additional reasons for investigating CFI as a plausible organizational model for larger groups, and should encourage future work to further explore how it could inspire new forms of decentralized and unscripted teamwork.
